# P89 Treatment-refractory New World cutaneous leishmaniasis misdiagnosed as recurrent cellulitis in a UK traveller returning from the Amazon

**DOI:** 10.1093/jacamr/dlag102.095

**Published:** 2026-06-26

**Authors:** Amina Khan, Vikram Mahadasa, Bakht Khan

**Affiliations:** University Hospital Southampton NHS Trust, Southampton, UK; University Hospital Southampton NHS Trust, Southampton, UK; University Hospital Southampton NHS Trust, Southampton, UK

## Abstract

**Background:**

Cutaneous leishmaniasis (CL) is a neglected tropical disease with diverse clinical presentations, often mimicking common bacterial skin infections. In non-endemic countries, lack of clinical familiarity frequently leads to delayed diagnosis, inappropriate antimicrobial therapy and increased morbidity. New World cutaneous leishmaniasis caused by *Leishmania (Viannia* subgenus) carries an additional risk of lymphocutaneous dissemination and mucosal involvement, making early recognition and appropriate management essential.

**Case presentation:**

A previously healthy man in his early 20s presented in the UK with progressive swelling of the left hand and fingers associated with a chronic ulcer on the dorsum of the left hand and nodular lesions extending up the left arm. Symptoms began 2 weeks after returning from a 5 week trip to the Amazon rainforest in Peru, with brief travel to Brazil, during which he sustained multiple insect bites. The lesion initially appeared as a small punctate wound and gradually evolved over several weeks into a deep ulcer with rolled edges, malodour, and serosanguinous discharge. Despite repeated presentations to primary care and multiple courses of antibiotics for presumed infected insect bite and recurrent cellulitis, there was progressive enlargement of the ulcer, reduced finger mobility, and development of painful nodules along the lymphatic drainage of the arm, consistent with sporotrichoid spread. Three months after symptom onset, he was referred to Plastic Surgery with concern for necrotizing infection, and a biopsy was obtained. He subsequently presented to Infectious Diseases due to diagnostic uncertainty. Examination revealed a 4 cm heavily exudative ulcer on the dorsum of the left hand with ipsilateral axillary and biceps lymphadenopathy. Microscopy of the biopsy demonstrated intracellular amastigotes, and PCR confirmed *Leishmania (Viannia)* subgenus. Serological testing for HIV, viral hepatitis, syphilis, and strongyloidiasis was negative. Flexible nasal endoscopy showed no evidence of mucosal involvement.

**Management and outcome:**

The patient was commenced on IV liposomal amphotericin B. However, treatment was complicated by stage 3 acute kidney injury after three doses, necessitating hospitalization, treatment interruption, and subsequent dose spacing under multidisciplinary guidance. Although partial wound improvement was observed, disease progression occurred with the development of new nodular lesions, and repeat biopsy remained PCR-positive, indicating treatment failure. The case was reviewed at a National Leishmaniasis Multidisciplinary Meeting, where re-treatment with IV meglumine antimoniate was recommended due to the Viannia subgenus and lymphocutaneous dissemination. He completed a 21 day course of meglumine antimoniate via a PICC line with close biochemical and ECG monitoring, which was well tolerated. Marked clinical improvement followed, with epithelization and healing of the primary ulcer and resolution of satellite lesions. No mucosal disease or recurrence was observed on follow-up.

**Conclusions:**

This case highlights the role of cutaneous leishmaniasis as a ‘great imitator’, particularly in non-endemic settings where chronic skin lesions are frequently attributed to bacterial infection alone. Failure to improve despite multiple antibiotic courses, gradual lesion evolution, and sporotrichoid lymphocutaneous spread should prompt consideration of CL and early biopsy. Species identification is critical, as therapeutic response and risk of complications vary by subgenus. Treatment is often challenging due to drug toxicity, emerging resistance, and the need for prolonged parenteral therapy, reinforcing the importance of multidisciplinary input. With increasing global travel, climate change, and expanding vector habitats, heightened clinical awareness is essential to prevent diagnostic delay, irreversible scarring, and long-term physical and psychological morbidity.
